# Corrosion Behavior and Mechanical Property of 5182 Aluminum/DP780 Steel Resistance Spot Welding Joints

**DOI:** 10.3390/ma17112472

**Published:** 2024-05-21

**Authors:** Xuan Shi, Sai Zhang, Tao Li, Xianming Meng, Congqian Cheng, Jibin Pei, Tieshan Cao, Jie Zhao

**Affiliations:** 1 School of Materials Science and Engineering, Dalian University of Technology, Dalian 116024, China; msenginsx@mail.dlut.edu.cn (X.S.); tieshan@dlut.edu.cn (T.C.); jiezhao@dlut.edu.cn (J.Z.); 2 China Automotive Technology and Research Center Co., Ltd., Tianjin 300300, China; zhangsai@catarc.ac.cn (S.Z.); litao2022@catarc.ac.cn (T.L.); 3 School of Railway Locomotive and Vehicle, Jilin Railway Technology College, Jilin 132299, China; peijb@sina.com

**Keywords:** aluminum/steel resistance spot welding joint, salt spray corrosion, interfacial compound, mechanical properties

## Abstract

Corrosion behavior is critical to the application of lightweight aluminum/steel joints using new resistance spot welding (RSW) technology. The study investigated the corrosion mechanism and the shear strength of RSW joints comprising 1.2 mm 5182 aluminum and 1.5 mm DP780 galvanized steel. Electrochemical corrosion tests were conducted on the base materials and various positions of the welds in a 3.5% NaCl solution. This result revealed that the corrosion susceptibility of the interfacial intermetallic compound (IMC) layer was not accelerated by the aluminum nugget because of the noble corrosion potential. Subsequently, the spray acceleration test was employed to investigate the corrosion mechanism. It is noteworthy that microcracks, as well as regions enriched with silicon and oxygen at the interface front, are preferential to corrosion during salt spray exposure, instead of the IMC layer. Moreover, the shear strength of the joints decreases with the reduction in the effective joint area after the salt spray exposure of the weld joints. This research systematically explored the corrosion behavior and its relationship with the mechanical properties of Al alloy/steel RSW joints.

## 1. Introduction

To reduce fuel and battery consumption in a cost- and environmentally effective way, the usage of lightweight and advanced materials in hybrid body structures is becoming a important strategy in the automotive industry [1,2]. Aluminum (Al) alloys and high-strength steels, as two typical materials, have huge differences in physical and metallurgical properties, leading to a challenge in establishing reliable joining techniques [3,4,5]. In many joining techniques, resistance spot welding (RSW) is one of the most promising candidates due to its high flexibility and efficiency [6,7]. The high tensile and fatigue properties of the Al alloy/steel joints can be achieved by using the optimized RSW joining technique [8,9,10]. However, the Al alloy/steel RSW joint is still susceptible to corrosion and joint strength degradation in an aggressive environment due to the complicated microstructure and intrinsic differences in the electrochemical corrosion properties at the joint interface [11,12]. Therefore, the corrosion behavior must be understood to ensure the reliable employment of the Al alloy/steel RSW technique.

Currently, the research on the corrosion behavior of Al alloy/steel joints has focused on the microstructure with different weld techniques and its evolution on the corrosion resistance. The self-piercing rivet (SPR) joints after salt spray tests showed galvanic coupling and crevice corrosion. The thickness of aluminum sheet was reduced and eventually cracked [13]. The surface-galvanized Zn coating is the most susceptible to corrosion due to its lowest corrosion potential. Thus, it protects the steel and interfacial phases as a sacrifice anode in various types of Al alloy/steel joints [14,15,16,17]. Besides the galvanized coating, the role of interfacial intermetallic compound (IMC) layers is more complicated and focused on by the previous research [15,16,17,18,19]. Lei et al. reported preferential corrosion of the interfacial Fe-Al IMC layer following the galvanized corrosion and thereafter changed the fracture model [17]. Considering the IMC thickness within several micrometers and the residual galvanized layer, it is difficult to achieve the intrinsic corrosion property of the IMC layer by general electrochemical test from the joint cross-section. After thoroughly cleaning out the residual zinc contamination and ensuring by EDX, higher corrosion resistance of the interfacial IMC layer was actually evidenced by Zhang et al. [20].

However, the corrosion of Al alloy/steel does not always occur in the interfacial IMC layer because of different microstructures and compositions at the interface front [18,19]. For example, Ma et al. reported the corrosion of Al near the IMC layer in 2A14 Al alloy/304 stainless steel friction joints [18]. Mahto et al. [19] found that the dissolution of the precipitated phase in the Al side improved the corrosion resistance of Al alloy/steel friction stir weld joints. Li et al. [16] found the intergranular corrosion of Al alloy instead of IMC layer corrosion. Recently, Dang et al. [21] reported that the formation of an interfacial Cu-rich layer at the AA2219/304 stainless steel joint accelerated the Al corrosion around the layer.

In fact, the interfacial microstructure is directly controlled by a specifically designed RSW joint method and thus involves the joint strength. The high fluence of heat input results in the excessive growth of brittleness in the IMC layer [22]. To overcome the small nugget and the formation of excessive brittleness in the IMC layer, a multi-ring domed electrode (MRDE) is designed and applied to achieve high joint quality [23,24,25]. Spontaneously, the corrosion behavior of the Al/steel RSW joint by using this new method is becoming interesting for the technique improvement from the view point of service property. Recently, Pan et al. reported the corrosion production at the overlap region of the RSW joint during the salt spray test and found the exitance of galvanic corrosion [26]. However, the corrosion mechanism at the joint interface and the strength degradation have not been reported on the new RSW technique by using MRDE. Particularly, the role of the IMC layer and the interface front on the corrosion behavior has not been clearly explored on the new RSW joint.

In this paper, 5182 aluminum alloy/DP780 steel RSW joint by using the MRDE were employed to investigate the electrochemical corrosion properties at the different localized regions of the joint in NaCl solution and corrosion behavior under a salt spray environment. The corrosion mechanism at the joint interface under the environment was focused based on detailed microstructure exanimation and the electrochemical corrosion results. The relationship between the corrosion behavior and shear strength was established based on the strength test and corrosion mechanical analysis.

## 2. Materials and Methods

### 2.1. Materials and Sample Preparation

Both DP780 steel and 5182 aluminum alloy were provided by the China Automotive Research Institute (Tianjin, China) for the preparation of dissimilar joint plates in resistance spot welding. The DP780 steel is HC420/780DPD + Z high-strength dual-phase steel plate with hot-dip galvanization. The 5182 aluminum alloy had been treated at T4 state, namely natural aging after solid solution treatment. The aluminum alloy plates in the size of 81 × 38 × 1.2 mm and the galvanized steel plates in the size of 81 × 38 × 1.5 mm were cut from the sheets. Table 1 presents the chemical compositions (in wt. %) of the two materials, as measured using optical emission spectroscopy.

**Figure 1:**
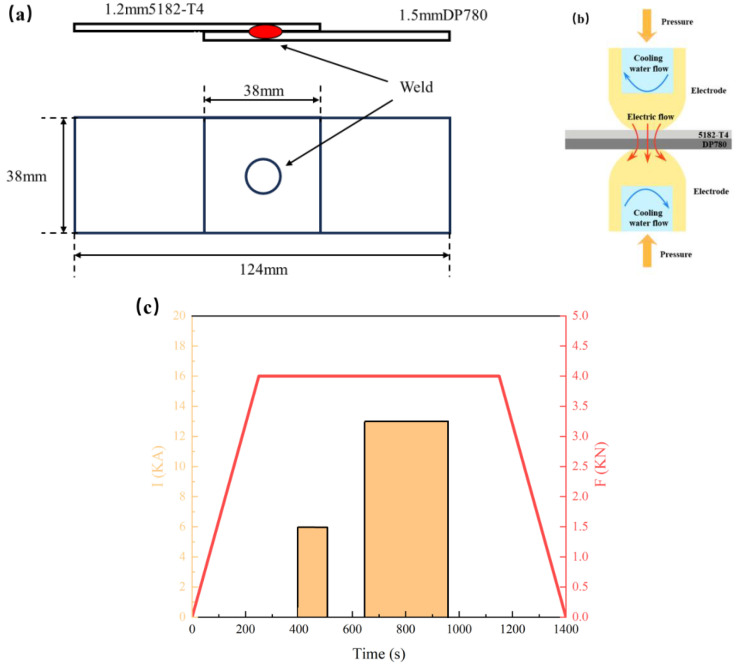
Schematic illustration of the RSW process: (**a**) overlap specimens and the welding position, (**b**) the schematic of resistant spot welding (RSW), and (**c**) welding schedule.

Overlap joints of the aluminum/steel were prepared by using RSW techniques (Centerline, Guangzhou, China). Figure 1a,b shows the schematic of the overlap specimens and the welding schedule during the RSW process. Aluminum alloy sheets and steel sheets were connected by resistance spot welding in a tensile shear configuration (Figure 1a). This was accomplished using a PLC-controlled 220 kVA MFDC (BEIYE, Taizhou, China) pedestal-type resistance spot welder operating at 1000 Hz. The same electrodes (MRD electrode [27]) were used on both sides of the aluminum alloy and steel sheets. The cooling rate of water was controlled at 2 gallons per minute. Other welding parameters are shown in Figure 1c.

### 2.2. Electrochemical Polarization Test

**Figure 2:**
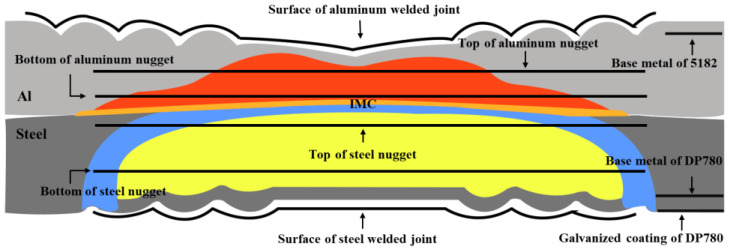
Schematic diagram of specimen selection location for the electrochemical test from the cross-section view (Figure 1b) of the 5182/DP780 RSW joint.

Different regions of the aluminum/steel RSW joints exhibit different corrosion features due to welding microstructure variation. The specimen locations from the cross-section of the joints are shown in Figure 2. Since the cross-sectional microstructure had been clearly examined, the different locations for the electrochemical test can be roughly estimated according to the thickness distance from the substrate surface or fracture surface and prepared by grinding the fracture samples. These fracture pieces were obtained after quasi-static tensile tests on the RSW joints by a uniaxial testing machine (SUSTCMT5205, SENS, Shenzhen, China) at a displacement rate of 3 mm/min. Ten layers were taken gradually from the metal surfaces to the interfacial IMC layer by grinding layer by layer. The biggest challenge during the preparation is for the sample at the IMC layer. The fracture specimens on the steel side were first chosen after the tensile test, and then a thin layer of residual aluminum was slowly and carefully ground by using 4000# sandpaper and polished. Since the composition of the IMC layer and residual aluminum is different, whether the IMC had been exposed can be identified by EDX. After cleaning and drying, the sample surfaces were sealed with 704 silicone glue, and the test areas were exposed for an electrochemical corrosion test.

Potentiodynamic polarization was conducted with a three-electrode measurement system in a CS310 electrochemical workstation and a Faraday shielded box. The three-electrode system was composed of the Ag/AgCl reference electrode (SSE) with saturated KCl solution, the counter Pt electrode, and the samples as work electrode. The potentiodynamic polarization was conducted in a 3.5 wt. % NaCl solution at 20 °C at a scanning speed of 0.5 mV/s, and after that the open-circuit potential was stabilized for 30 min. Most polarization scanning began between −600 mV and −500 mV versus the open circuit potential, and continued until the anode region could express the characteristics of corrosion. However, the scanning for galvanized coating on the steel surface started from −1.6 V versus the SSE due to its low open circuit potential. Each location of the joints was measured three times.

### 2.3. Salt Spray Corrosion and Mechanical Test

A neutral salt spray test was conducted in a JD-60 Salt Spray Tester according to the ISO-9227 standard [28]. The specimen was placed at a 30-degree angle to the vertical datum line and the steel was at the bottom of the environment. The brine was a solution of sea salt grains with a pH value between 6.5 and 7.2, containing 50 g/L of salt. The temperature in the salt spray chamber maintained a consistent 35 ± 2 °C, and five exposure periods were particularly examined: 72 h, 240 h, 480 h, 720 h, and 1080 h. The static tensile shear test was conducted on both the original specimens and after salt spray exposure by using an electronic testing machine (Instron 5982, Instron, Norwood, MA, USA) at a speed of 2 mm/min.

Based on the corrosion of the fracture characterization after different salt spray exposure times, the corroded area shown in the yellow dashed area of fracture morphology after different times salt spray exposure was determined. The image analysis program in Matlab R2021a was utilized to calculate the corrosion area at the fracture of the RSW joints.

### 2.4. Microstructure Examination

The cross-sectional microstructure of the joint and the fracture morphology after the tensile test were observed by using a field emission gun scanning electron microscope (SEM; Crossbeam SUPRA 55, Zeiss, Jena, Germany). The specimens for the cross-sectional observation were cut from the center of the weld joints. After grinding and polishing, the specimens were etched by sodium hydroxide solution and alcohol nitrate solution. The element mapping and surface scanning at the localized interface were performed by an electron probe micro analyzer (EPMA; JXA-8530F PLUS, JEOL Ltd., Tokyo, Japan).

## 3. Results and Discussion

### 3.1. Microstructure Characteristics of RSW Joints

**Figure 3:**
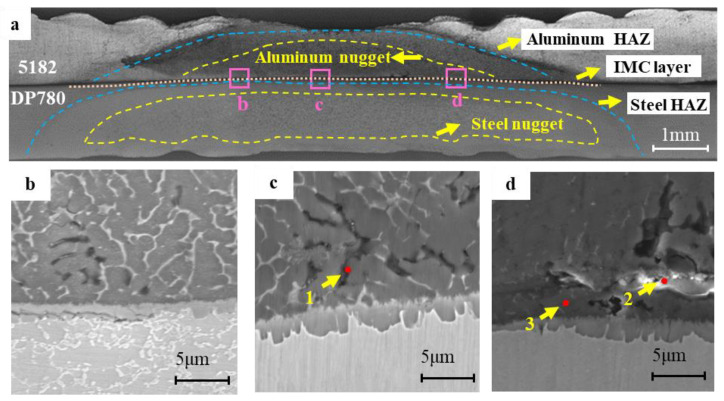
Microstructure characteristic of 5182/DP780 RSW joint, (**a**) the cross-sectional microstructure of the RSW joint (**b**) the microstructure of the interface at position b, (**c**) the microstructure of the interface at position c, and (**d**) the microstructure of the interface at position d.

Figure 3 shows the original cross-sectional microstructure of the RSW joint and the composition at the selected interface front is shown in Table 2. The RSW joints are composed of a steel nugget, the heat-affected zone, the interfacial layer, and an aluminum nugget. In the center of the nugget, the tongue-like interfacial intermetallic compound layer was observed between the aluminum nugget and the steel (Figure 3c), which plays an important role in the metallurgical combinations of steel and aluminum [29]. At the interface front in the aluminum nugget, significant dendrite microstructure formed whereas the shrinkage cavity richened in Si and O, which seems visible on the aluminum side as pointed out at position 1 in Figure 3c. The presence of the shrinkage might negatively impact the mechanical properties of the joints. At one side of the central nugget, the thickness of the IMC layer decreased and microcracks existed along the interface IMC layer in several joints, as shown in Figure 3b. The microcrack might be attributed to the induced high stress during the fast cooling after welding. In most joints near the central nugget, a very narrow band with a high content of oxygen and several low content of light metal elements emerged at the front of the interfacial intermetallic compounds on the aluminum side as pointed out at position 2 in Figure 3d, indicating a probable existence of oxide. The oxide at the interface front may be due to the ruptured oxide of the aluminum surface entrapped in the interfacial compound during the welding process [30].

**Figure 4:**
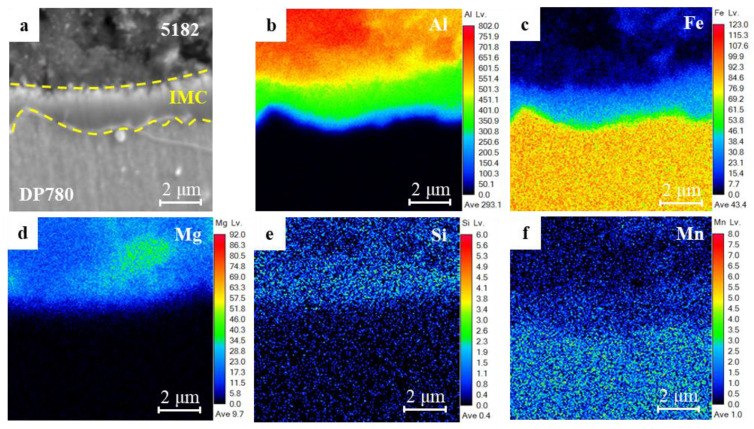
Distribution of major alloying elements in the interface of the 5182/DP780 RSW joint as determined by the EPMA element mapping: (**a**) microstructure characteristic of the IMC layer, (**b**) Al element, (**c**) Fe element, (**d**) Mg element, (**e**) Si element, (**f**) Mn element.

Figure 4 shows the EPMA element distribution of the IMC layer at the nugget center of the 5182 aluminum/DP780 steel RSW joint. Compared to the Al nugget (redness region in Figure 4b), two regions in green and blue seem to be observed at the interface IMC layer. Such regions are coincidental to the regions in blue and green of Fe mapping in Figure 4c, respectively. The EPMA element mapping results in this paper were consistent with the previous research about the elemental distribution of steel/aluminum IMC by EDS and the EBSD phase map [31,32]. According to the atomic ratio [33,34,35,36,37,38], the two layers of the interfacial IMC are FeAl_3_ and Fe_2_Al_5_, respectively. The Mg is found only on the aluminum side (Figure 4d). A slight amount of Mn element seems to be visible in Figure 4f, while slight enrichment in Si element can be carefully identified in Figure 4e. These elements play a crucial role in controlling the growth of interfacial intermetallic compounds [39,40,41,42,43].

### 3.2. Potentiodynamic Polarization

**Figure 5:**
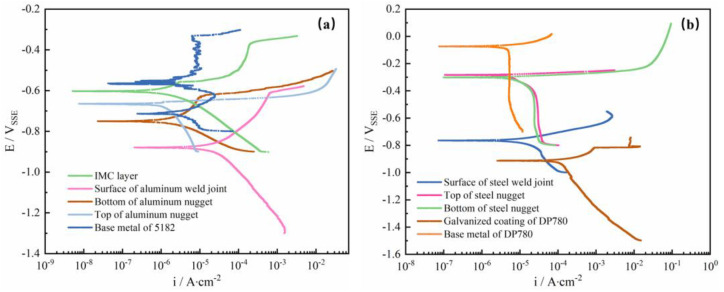
Potentiodynamic polarization curves in a 3.5 wt. % NaCl solution for different localized layers within the RSW joints of 5182 aluminum alloy and DP780 galvanized steel from the fracture samples. (**a**) The 5182 aluminum alloy side; (**b**) the DP780 galvanized steel side.

The potentiodynamic polarization curves of the different zones of the RSW joints presented in Figure 2 are shown in Figure 5. For the samples from the fracture of the aluminum alloy side in Figure 5a, the investigated aluminum nugget and surface of weld joint exhibit distinct features of passivation and breakdown [44]. Such breakdown is also evident in the IMC layer sample [45]. The presence of passivation and breakdown might be expected in the aerated salt solution as the aluminum alloy is a passive metal, particularly when surface oxide film might form on the Al alloy during the glue mounting for test after the grinding preparation. For the base metal of Al alloy, two peaks appeared in the polarization curves, indicating the instability of the surface oxide film. For the samples from the fracture of the steel side in Figure 5b, polarization behavior with significant anodic active dissolution and cathodic diffusion control are notable [46,47]. Table 3 lists the primary parameters of corrosion calculated from the polarization curves. The aluminum alloy exhibits passivation properties, except the top surface of the aluminum nugget. The IMC curve drifts to the upper left corner, indicating a higher corrosion potential compared to the nugget of aluminum alloy, as shown in Figure 4a. The steel side exhibits activated corrosion. The galvanized layer has the lowest corrosion potential and highest corrosion current density. Meanwhile, the highest corrosion potential and a low corrosion current density were evident on the base metal of DP780.

From Figure 5 and Table 3, the sequence of E_corr_ in each region is as follows: the base metal of DP780 > the top of steel nugget > the bottom of steel nugget > the base metal of 5182 > IMC layer > the top of aluminum nugget > the bottom of aluminum nugget > the surface of steel weld joint > the surface of aluminum weld joint > the galvanized coating of DP 780. Galvanic corrosion may occur between different regions [48]. From Table 3, when the joints are immersed in the electrolyte, the galvanized layer as sacrificial anode spontaneously corrodes first to protect the steel due to its most negative corrosion potential. Interestingly, the interfacial IMC layer presents relatively good corrosion resistance. And, its corrosion seems not to be accelerated by the aluminum nugget because of the noble corrosion potential. This result is different from the general consideration that the IMC layer was mostly susceptible to corrosion damage due to the galvanic effect [18], but consistent with our previous research [20]. Such a different result may be due to the careful sample preparation from the fracture surface and the influence of the aluminum nugget could be avoided after the EDX reassurance. Thereafter, the intrinsic corrosion property of the IMC layer can be explored. Between the IMC layer and the bottom nugget, the bottom of the aluminum nugget has more negative potential in the galvanic couple. So, the interface front is contrarily more susceptible to accelerated corrosion compared to the interfacial IMC layer.

### 3.3. Salt Spray Corrosion Test

**Figure 6:**
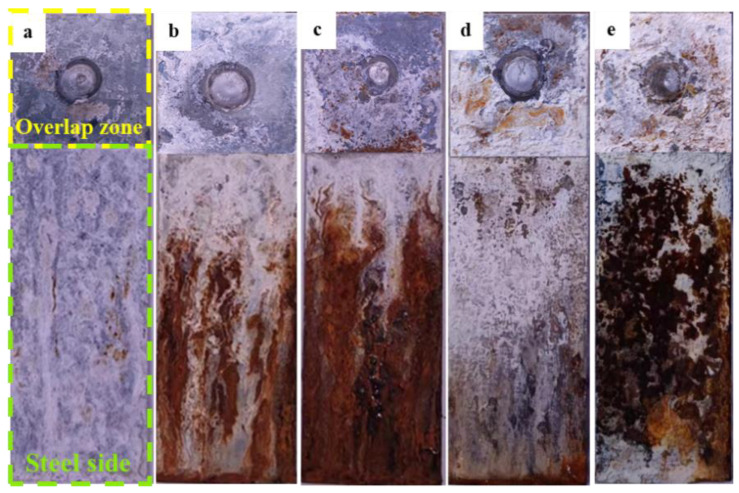
Corrosion morphologies of the overlap zones on the steel side after salt spray exposure at different times (**a**) 72 h, (**b**) 240 h, (**c**) 480 h, (**d**) 720 h, and (**e**) 1080 h.

Figure 6 displays the morphologies of the overlap zone of the aluminum/steel RSW specimen after salt spray exposure at different times. The overlap zones experience a lower degree of corrosion compared to the base steel. The overlap zone was covered by white corrosion products at the beginning of the salt spray test. With extended salt spray time, the corrosion spreads from the lower left of the overlap area to the center of the weld joint. The white corrosion products seem to change into rust-red corrosion products. Salt spray can enter the overlap area through the welded seams. With exposure time increasing, salt spray accumulates into salt liquid, then flows down the steel side, eventually causing severe corrosion of the steel.

**Figure 7:**
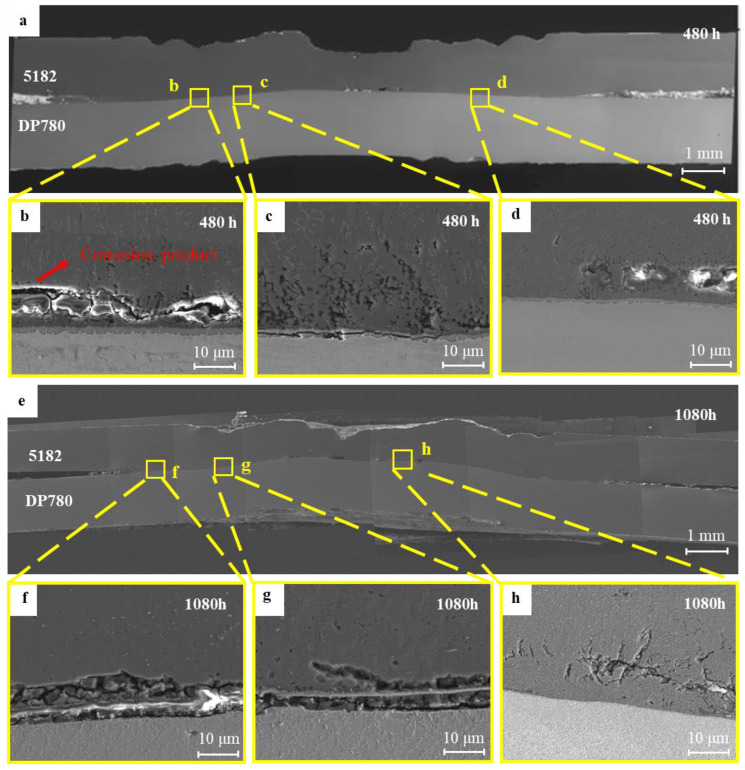
Cross-sectional microstructure of 5182/DP780 RSW joint after salt spray exposure of different times: (**a**) macrostructure of cross-section after salt spray exposure of 480 h; (**b**) microstructure at position b; (**c**) microstructure at point c; (**d**) microstructure at point d; (**e**) macrostructure of cross-section after salt spray exposure of 1080 h; (**f**) microstructure at point f; (**g**) microstructure at point g; (**h**) microstructure at point h.

To explore the corrosion behavior on the interface, the samples after salt spray exposure of 480 h and 1080 h were chosen, and their interfacial cross-sectional microstructures are shown in Figure 7. After salt spray exposure of 480 h, bright white corrosion products accumulated on the overlap region between the aluminum alloy and the steel, as shown in Figure 7a. From the high magnification on positions b and d in Figure 7a, corrosion products accumulated at the interface front of the aluminum alloy, but corrosion of the IMC layer was not found. This result further gives rise to the high corrosion sensitivity at the interface front close to the aluminum nugget. After salt spray exposure of 1080 h, severe corrosion along the microcracks and interface front was apparently observed as shown in Figure 7f–h. According to Figure 7h, corrosion developed towards the nugget center of the joint along the original microcracks on the interface front of the aluminum nugget side. Nevertheless, the residual of the IMC layer in Figure 7f,g still gives evidence of its intrinsic higher corrosion resistance compared to the interface front of the aluminum nugget.

**Figure 8:**
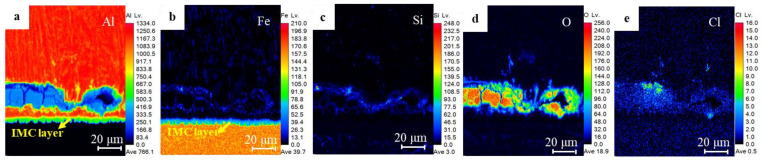
Distribution of element mappings at position b in Figure 7: (**a**) Al element, (**b**) Fe element, (**c**) Si element, (**d**) O element, (**e**) Cl element.

**Figure 9:**
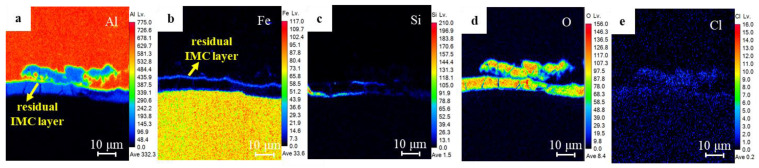
Distribution of element mappings at position g in Figure 7: (**a**) Al element, (**b**) Fe element, (**c**) Si element, (**d**) O element, (**e**) Cl element.

Figure 8 and Figure 9 show the distribution of major alloying elements at position b for the sample after the 480 h salt spray test and position g for the sample after the prolonged 1080 h test in Figure 7, respectively. From Figure 8a,b, the interface Al element region in green coincides with the interface Fe element region in blue, but without the O accumulation in Figure 8d, indicating the IMC layer has not been corroded. Instead, the region enriched in Si, O, and Cl elements near the interface (Figure 8c–e) is in accord with the blue region in the Al nugget in Figure 8a, indicating the preferential corrosion along the interface front rather than at the IMC layer. After the prolonged 1080 salt spray test, the accumulation of Si, O, and Cl (Figure 9c–e) coincided with the blue region of the Al element in Figure 9a. Interestingly, the existence of the IMC layer can still be clearly identified from Figure 9a,b. These corrosion regions in Figure 8 and Figure 9 coincide with the microcracks and Si-enriched region at the interface front in Figure 3, indicating the corrosion development along the microcracks and regions with an enrichment of Si and O near the interface front of Al nugget.

### 3.4. Fracture Characteristics and Mechanical Properties

**Figure 10:**
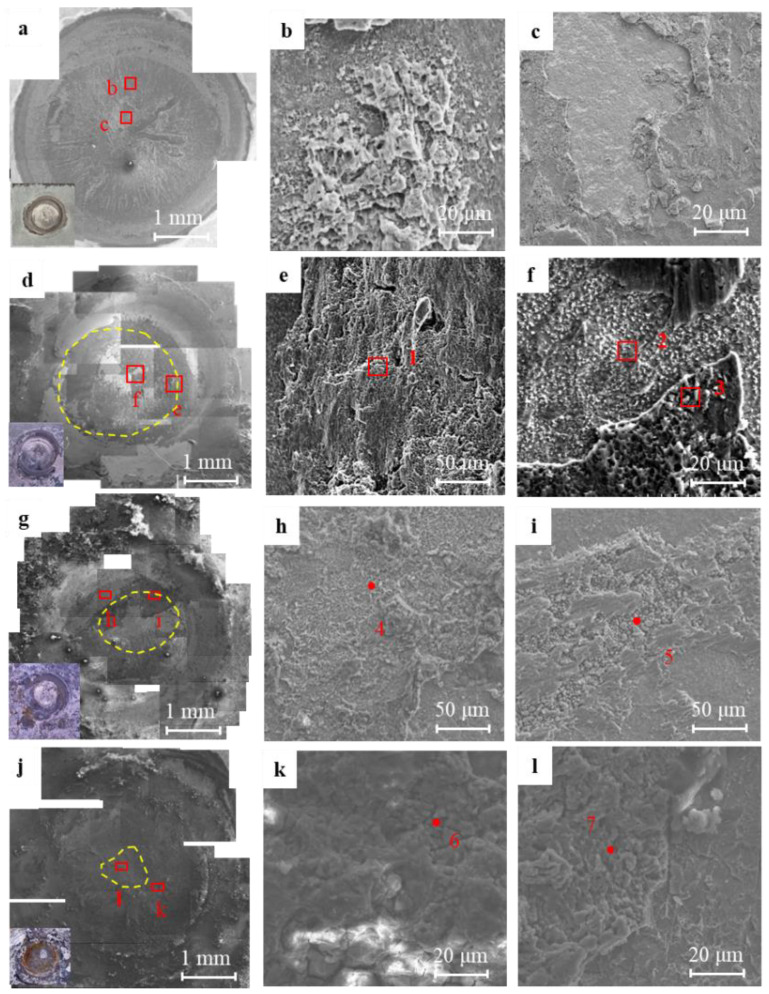
Fracture morphology of 5182/DP780 RSW joint after salt spray exposure of different times (**a**) macro-morphology without salt spray; (**b**) micro-morphology at point b; (**c**) micro-morphology at point c; (**d**) macro-morphology after exposure of 72 h; (**e**) micro-morphology at point e; (**f**) micro-morphology at point f; (**g**) macro-morphology after exposure of 480 h; (**h**) micro-morphology at point h; (**i**) micro-morphology at point i; (**j**) macro-morphology after exposure of 1080 h; (**k**) micro-morphology at point k; (**l**) micro-morphology at point l.

Figure 10 shows the fracture morphology of the RSW joints after the salt spray exposure. The specimen after the exposure of 72 h fractured similarly to the original specimen, with a nugget fracture mode in Figure 10a,d. The surface is relatively flat, and the center area of the nugget exhibits the white metal of the aluminum side. Table 4 presents the EDS results corresponding to the locations of the fracture morphology in Figure 10. The composition analysis at position 1 and position 2 showed an aluminum alloy. The composition of dimple morphology at position 3 was close to that of the steel side.

According to the fracture morphology after the exposure of 480 h, the fracture mode of the specimen is still a nugget fracture. The corrosion of the interface front on the aluminum side obviously takes place at the weld nugget’s edge, with the low content of the Al element. And, a small amount of Zn and Cl elements are present in the aluminum/iron oxide at position h of the weld nugget edge. The EDS indicates that positions h and k are the special locations with high oxygen content. Such results are consistent with the corrosion area shown by EPMA in Figure 8 and Figure 9. Position i was composed mainly of Al element with a minor quantity of oxygen. This suggests that the center of the weld nugget did not experience significant corrosion after the salt spray exposure of 480 h. The presence of oxygen may be due to the shrinkage cavity defect in the center of the weld nugget (according to Figure 3c).

After salt spray exposure of 1080 h, the corrosion area at the edge of the weld nugget continues to expand toward the center of the weld nugget. Finally, the bright white area in the center of the weld nugget was uncorroded according to the composition at position 7.

**Figure 11:**
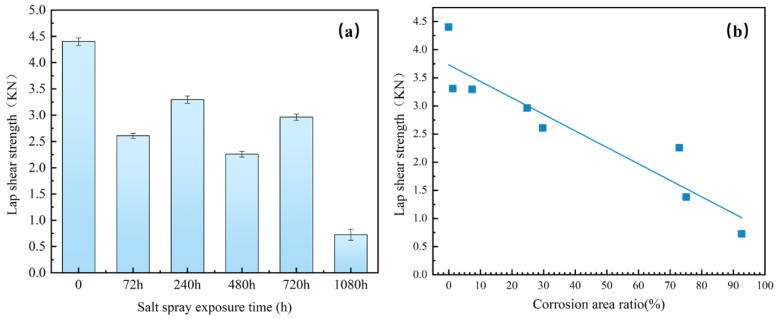
Lap shear strengths of the welded joints: (**a**) relationship between the lap shear strength and salt spray exposure time; (**b**) relationship between the lap shear strength and the ratio of corrosion area.

**Figure 12:**
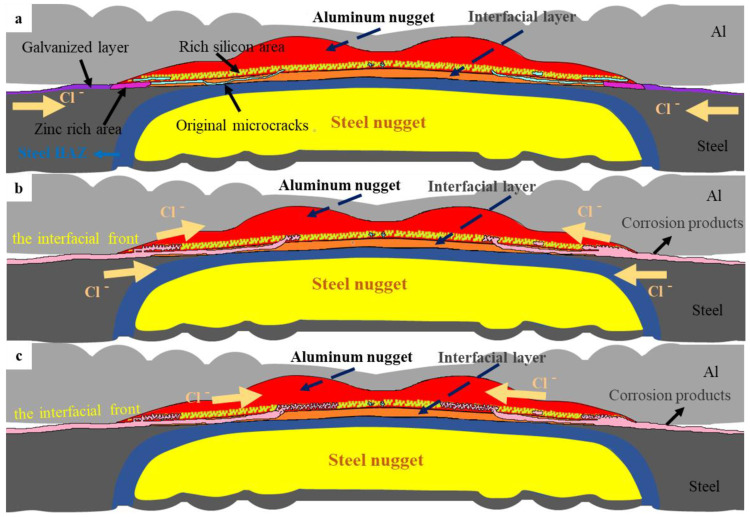
Corrosion model of 5182/DP780 RSW joint after salt spray exposure: (**a**) original RSW joint, (**b**) the RSW joint after short term of salt spray exposure, (**c**) the RSW joint after long term of salt spray exposure.

Figure 11 shows the relationship between the maximum tensile shear force and the corrosion area ratio. The shear strength of the joints decreases as the corrosion area of the weld joint increases. The corrosion model of the 5182/DP780 RSW joint after salt spray exposure is formulated in three stages based on the aforementioned findings, referring to Figure 12. During salt spray exposure, chloride attacks the joints. As the sacrificial anode protection, the galvanized layer first undergoes corrosion according to Figure 12a. Then, corrosion appears in the zinc-rich area along the edge of the overlap region. Afterwards, chloride continues to attack through the original microcracks on the interfacial front on the aluminum side, as illustrated in Figure 12b. Additionally, the previously affected region exhibits accumulations of corrosion products. With the prolongation of salt spray exposure time, the corrosion spreads to the front interface of Si enrichment on the aluminum side. Eventually, corrosion further develops along the nugget on the aluminum, according to Figure 12c. The joint corrosion observed in this study exhibits a significant inclination towards Si-rich corrosion. The corrosion on the front interface of the aluminum side degrades the mechanical properties of the joint.

## 4. Conclusions

In summary, the corrosion behavior and mechanical properties of the resistance spot welding joints of 5182/DP780 by using multi-ring domed electrodes were systematically investigated by microstructure observation, electrochemical corrosion test, and salt spray test. The main conclusions are as follows:

(1) According to microstructure observation, the joint between 5182 and DP780 is bonded by the formation of FeAl intermetallic compound layer. Si enrichment and a few oxidation defects were observed at the interface front on the aluminum nugget side.
(2) The experimental result from the polarization test and salt spray test gives evidence to the fact that the interfacial compound layer was not susceptible to preferential corrosion due to its relative noble corrosion potential in the interfacial galvanic couple.
(3) During the salt spray test, the corrosion of the joint initially occurred in the galvanized layer of the overlap region and then developed towards the nugget center of the joint along the original microcracks and the defects on the interface front of the aluminum nugget side. Such a result was consistent with the accelerated corrosion susceptibility of the aluminum nugget by anodic galvanic effect with a coupled interfacial IMC layer in the polarization test. According to the mechanical property test, the tensile shear strength decreased with an increase in the corroded area of the weld joint.

## Figures and Tables

**Table 1:** Chemical composition of the material (weight%).

Element	C	Al	Fe	Mg	Cu	Mn	Si	Cr	Zn	Mo	S	P
5182-T4	-	94.81	0.17	4.53	0.04	0.22	0.19	0.03	0.01	-	-	-
DP780	0.112	0.387	97.02	-	-	1.92	0.08	0.27	-	0.2	0.001	0.01

**Table 2:** Chemical composition at positions 1–3 in Figure 3 (wt. %).

Element	Al	Fe	O	Mn	Si	Mg	Cu	Cr	Zn
1	86	0.4	8.9	0.1	0.8	3.1	0.4	---	0.3
2	18	3.6	64	---	4.4	5.7	---	---	4.3
3	92	0.4	2.8	---	0.04	4.7	---	---	0.06

**Table 3:** Electrochemical parameters of different layers/regions in 5182 aluminum alloy/DP780 galvanized steel resistance spot welding joints.

Region	Bottom of Al Nugget	Top of Al Nugget	Surface of Al Weld Joint	Base Metal of 5182	IMC Layer	Galvanized Coating of DP 780	Base Metal of DP780	Surface of Steel Weld Joint	Bottom of Steel Nugget	Top of Steel Nugget
E_corr_/V	−0.746	−0.672	−0.881	−0.573	−0.603	−0.913	−0.074	−0.767	−0.303	−0.282
i_corr_/ (A/cm^2^)	2.1 × 10^−6^	1.7 × 10^−6^	1.7 × 10^−5^	7.5 × 10^−6^	2.0 × 10^−6^	2.1 × 10^−4^	8.7 × 10^−6^	9.7 × 10^−6^	9.1 × 10^−6^	5.2 × 10^−6^

**Table 4:** Chemical composition at positions 1–7 in Figure 11 (wt. %).

Element	Al	Fe	O	Mg	Mn	Zn	Cl
1	95	1.5	0.7	2.8	-	-	-
2	91	4.7	0.6	2.8	0.9	-	-
3	1.7	96	-	-	2.3	-	-
4	14	11	64	0.3	-	5.7	5
5	89	2	3	6	-	-	-
6	31	0.5	64	4.5	-	-	-
7	92	-	-	8	-	-	-

## Data Availability

Data are contained within the article.
